# Iroquois homeobox transcription factor (*Irx5*) promotes G_1_/S-phase transition in vascular smooth muscle cells by CDK2-dependent activation

**DOI:** 10.1152/ajpcell.00293.2015

**Published:** 2016-05-11

**Authors:** Dong Liu, Vaishnavi Pattabiraman, Methode Bacanamwo, Leonard M. Anderson

**Affiliations:** ^1^Cardiovascular Research Institute, Morehouse School of Medicine, Atlanta, Georgia;; ^2^Department of Physiology, Morehouse School of Medicine, Atlanta, Georgia; and; ^3^Department of Medicine, Morehouse School of Medicine, Atlanta, Georgia

**Keywords:** *Irx5*, vascular smooth muscle cell, DNA synthesis, cell cycle, cyclin-dependent kinase 2, apoptosis

## Abstract

The Iroquois homeobox (*Irx5*) gene is essential in embryonic development and cardiac electrophysiology. Although recent studies have reported that IRX5 protein is involved in regulation of the cell cycle and apoptosis in prostate cancer cells, little is known about the role of IRX5 in the adult vasculature. Here we report novel observations on the role of IRX5 in adult vascular smooth muscle cells (VSMCs) during proliferation in vitro and in vivo. Comparative studies using primary human endothelial cells, VSMCs, and intact carotid arteries to determine relative expression of *Irx5* in the peripheral vasculature demonstrate significantly higher expression in VSMCs. Sprague-Dawley rat carotid arteries were subjected to balloon catherization, and the presence of IRX5 was examined by immunohistochemistry after 2 wk. Results indicate markedly elevated IRX5 signal at 14 days compared with uninjured controls. Total RNA was isolated from injured and uninjured arteries, and *Irx5* expression was measured by RT-PCR. Results demonstrate a significant increase in *Irx5* expression at 3–14 days postinjury compared with controls. *Irx5* genetic gain- and loss-of-function studies using thymidine and 5-bromo-2′-deoxyuridine incorporation assays resulted in modulation of DNA synthesis in primary rat aortic VSMCs. Quantitative RT-PCR results revealed modulation of cyclin-dependent kinase inhibitor 1B (*p27*^*kip1*^), E2F transcription factor 1 (*E2f1*), and proliferating cell nuclear antigen (*Pcna*) expression in *Irx5*-transduced VSMCs compared with controls. Subsequently, apoptosis was observed and confirmed by morphological observation, caspase-3 cleavage, and enzymatic activation compared with control conditions. Taken together, these results indicate that *Irx5* plays an important role in VSMC G_1_/S-phase cell cycle checkpoint control and apoptosis.

proliferation of vascular smooth muscle cells (VSMCs) is important during vascular remodeling in atherosclerosis and restenosis (4, 36). Therapeutic strategies using various gene delivery modalities to impede cell cycle initiation and progression or promote apoptosis in VSMCs have been attempted to reduce postinjury intimal hyperplasia (10, 35).

Homeobox genes encode a family of transcription factors characterized by a 60-amino acid conserved DNA-binding motif known as the homeodomain (43). Despite their well-known effects during embryonic development, some homeobox genes are involved in pathological states such as the development of cancer (1) and atherosclerosis and restenosis after angioplasty (17, 20, 25, 26). Iroquois homeobox (*Irx*) genes are evolutionarily conserved from sponges to humans (32). The Iroquois gene family belongs to the TALE (3-amino-acid loop extension) superclass of atypical homeodomain-containing proteins that also contains a class-defining nine-amino acid motif termed the IRO box of unknown function (8). Six Iroquois (*Irx1-6*) genes in mammals and lower organisms have been shown to be involved in development (2, 19, 30, 33).

IRX5 protein has been characterized in the developing mouse embryo (6, 12, 14). In situ hybridization studies revealed the expression pattern of *Irx5* in the developing heart and nervous system in mice and lower organisms (13, 14). IRX function is highly dependent on cell type and context. Studies using *Irx5* null mice indicate that *Irx5* is required for retinal cone bipolar cell development and formation of the cardiac ventricular repolarization gradient by direct repression of Kv4.2 K^+^ channel expression (11, 15). Previous clinical studies report that *Irx5* expression is elevated in ventricles of patients with dilated cardiomyopathy (3). Studies in *Xenopus* embryos revealed that *Irx5* is positively regulated by another homeodomain transcription factor, *Hoxb4*, that can activate the apoptotic pathway (28, 40). Transient knockdown of *Irx5* resulted in cell cycle arrest in the G_2_/M phase and subsequent apoptosis in the hyperproliferative human prostate cancer cell line LNCaP in a vitamin D_3_-dependent manner (31). Thus these cumulative observations imply that *Irx5* might function as a cell growth regulator in adult VSMCs during proliferative vasculopathic disease progression.

Here we report that *Irx5* is expressed in human and murine VSMCs and that *Irx5* expression is significantly increased in response to mitogenic stimulation. The presence of IRX5 protein was elevated in VSMCs in the neointima after balloon injury in rat carotid arteries. Furthermore, enforced expression of *Irx5* results in loss of G_1_/S-phase checkpoint control, elevation of DNA synthesis activity, and reduced cell growth rate, as well as apoptosis following S-phase arrest. Thus these results suggest that *Irx5* may partially govern adult VSMC fate in the context of proliferative vascular disease.

## MATERIALS AND METHODS

### 

#### Rat carotid artery balloon injury.

All animal studies and procedures were approved by the Institutional Animal Care and Use Committee of the Atlanta University Center. Male Sprague-Dawley rats (350–400 g body wt; Charles River Labs, Raleigh, NC) were anesthetized with ketamine (80 mg/kg) and xylazine (6 mg/kg) and subjected to balloon injury, as previously described (29). Briefly, an F2 Fogarty catheter was inserted into the carotid artery, inflated, and pulled back and forth six times to denude the vessel. Animals were euthanized, and thoracotomies were performed. Carotid arteries were harvested and snap-frozen or embedded in paraffin at the indicated times for total RNA isolation and immunohistochemical analysis, as previously described (29).

#### Tissue isolation, processing, and immunostaining.

At the indicated times, rat carotid arteries were perfused with PBS for 5 min, and a 2-cm section of carotid artery distal to the aorta was excised and incubated overnight in 10% buffered formalin solution. Segments of the artery were cut into eight serial 5-μm-thick cross sections at 0.15-mm intervals, as previously described (29). Total RNA from carotid arteries was isolated, and quantitative RT-PCR was performed as described elsewhere (29). For immunohistochemical analysis, sections were rehydrated, blocked with normal serum and 0.01% Triton X-100 in PBS, and incubated with anti-IRX5 primary antibody (1:600 dilution; PAI-17056, Affinity Bioreagents, Golden, CO). Nonimmune IgG (1:600 dilution) was used as a negative control. Sections were incubated with biotinylated secondary antibody and developed with avidin-biotin-peroxidase reagent and then with 3,3′-diaminobenzidine (DAB Substrate Kit for Peroxidase, Vector Laboratories, Burlingame, CA) for detection. Cell nuclei were counterstained with hematoxylin, and immunohistochemical images were captured using an Olympus BX60 microscope at ×40 magnification.

#### Cell culture.

Primary rat aortic smooth muscle cells (RASMCs) were obtained from Cell Applications (San Diego, CA). Human aortic smooth muscle cells (HASMCs) and human umbilical vein endothelial cells (HUVECs) were purchased from Cambrex (Baltimore, MD). HUVECs were maintained in endothelial cell growth medium. HASMCs were maintained as previously described (29). Low-passage (*1–6*) RASMCs were cultured in growth medium: DMEM-F12 medium supplemented with 10% FBS and antibiotics. For all cell cycle studies, RASMCs were made quiescent in serum-deprived medium containing DMEM-F12 medium + 0.1% FBS for 48 h.

#### Real-time PCR analysis.

Total RNA isolation, cDNA reverse transcription, and quantitative RT-PCR were performed as previously described (29). PCR products were analyzed by electrophoresis on 2% agarose gels containing ethidium bromide (Thermo Fisher Scientific, Grand Island, NY) to confirm primer specificity. All primers were obtained from Eurofins MWG Operon (Huntsville, AL). Primer sequences and product sizes are provided in Table 1.

**Table 1.** Primer sequences for real-time PCR studies

		Primer		
Gene	GenBank Accession No.	Forward (5′-3′)	Reverse (5′-3′)	Product Length, bp
Human *Irx5*	NM_005853.4	ccgccgccgccttctcctcgta	tggcgtcccttgtggcgttcttcc	150
Human *β-actin*	NM_001101.2	gctcgtcgtcgacaacggct	caaacatgatctgggtcatcttctc	353
Rat *Irx5*	NM_001030044.1	cgccggcgcctcaagaaaga	gacccgcatcctccgccagac	364
Rat *β-actin*	NM_031144.1	cctaaggccaaccgtgaaaagatg	gtcccggccagccaggtccag	219
Rat *E2f1*	XM_230765.4	agcgcctggcctatgtgacctg	tcgatggggccttgtttgctctta	160
Rat *Pcna*	NM_022381.2	cacgtatatgccgggacctta	cagtggagtggcttttgtgaa	230
Rat *Gadd45β*	NM_001008321.1	gaggcggccaaactgatgaatgtg	ggtctcgggcctcgtttgtgc	226
Rat *Gadd45γ*	NM_001077640.1	acgagtccgccaaagtcctgaatg	gtctcccacgcgcacgatgtc	161
Rat *p27*^*kip1*^	NM_031762.2	gcggtgccttcaattgggtctca	cgggcttcttgggcgtctgctc	232

*Irx*, Iroquois homeobox transcription factor; *E2f1*, E2F transcription factor 1; *pcna*, proliferating cell nuclear antigen; *Gadd*, growth arrest and DNA damage; *p27*^*kip1*^, cyclin-dependent kinase inhibitor 1B.

#### Construction of recombinant adenovirus vectors.

Replication-deficient adenovirus vectors were constructed using Gateway technology (Thermo Fisher Scientific), as previously described (29). Briefly, Ad/Irx5 was generated by using the full-length mouse *Irx5* cDNA (GenBank accession no. NM_018826) from the plasmid pYX-Asc/Irx5 (Thermo Fisher Scientific). To facilitate detection of exogenous IRX5, the fusion protein (IRX5-V5) was expressed with addition of Tag-On-Demand suppressor supernatant (Thermo Fisher Scientific). Recombinant adenoviral vector expression cassettes were confirmed by restriction enzyme mapping and PCR. Ad/LacZ was used a negative control for these studies.

To create the Ad/microRNA (miR)-Irx5 vector, the expression clone pcDNA6.2-GW/EmGFP-miR-Irx5 was generated by ligation of the linearized vector cDNA6.2-GW/EmGFP-miR with oligonucleotides designed by using BLOCK-iT RNAi Designer (Thermo Fisher Scientific). Based on rat *Irx5* (GenBank accession no. NM_001030044.1), we tested three microRNA target sequences that start at positions 309, 387, and 416, respectively. The target sequence starting at position 309, 5′-ACCTCTGGGCTCCTATCCTTA-3′, was used for this study. pAd/miR-Irx5 was created by two steps of recombination with the entry clone pDONR221 and pAd/CMV/V5-DEST plasmids.

Each insertion was verified by restriction endonuclease analysis and sequencing. The replication-deficient adenovirus vectors Ad/Irx5, Ad/LacZ, Ad/miR-Irx5, and Ad/miR-Neg were produced by transfection of 293A cells with linearized and purified pAd/Irx5, pAd/LacZ, pAd Ad/miR-Irx5, and pAd/miR-Neg, respectively. Adenoviruses were then purified by cesium chloride gradient centrifugation, dialyzed in a Float-A-Lyzer (Spectrum Laboratories, Rancho Dominguez, CA) against PBS, and titered using an Adeno-X rapid titer kit (Clontech Laboratories, Mountain View, CA). Ad/LacZ and Ad/miR-Neg were used as negative controls.

#### Western immunoblotting.

RASMCs were lysed by addition of protein extraction reagent (Pierce) with protease inhibiter cocktail (Sigma). The concentration of protein in cell lysates was determined using protein assay dye reagent (Bio-Rad Laboratories, Hercules, CA). Monoclonal V5-horseradish peroxidase antibody (Thermo Fisher Scientific) was used at 1:5,000 dilution, polyclonal cleaved caspase-3 antibody at 1:100 dilution, and polyclonal caspase-3 antibody (EMD Millipore, Billerica, MA) at 1:1,000 dilution. P27^Kip1^ (1:1,000 dilution), GAPDH (1:2,000 dilution), E2F transcription factor 1 (E2F1, 1:300 dilution), and proliferating cell nuclear antigen (PCNA, 1:2,000 dilution) antibodies were obtained from Cell Signaling Technology (Beverly, MA). Growth arrest and DNA damage (GADD45β and GADD45γ) antibodies were obtained from Abcam (Cambridge, MA). Protein bands were visualized with SuperSignal chemiluminescence substrate (Thermo Fisher Scientific).

#### Determination of DNA synthesis.

[^3^H]thymidine incorporation was determined as previously described with modifications (29). RASMCs were transduced with the indicated adenovirus vector [multiplicity of infection (MOI) = 200] for 8 h and replaced with growth arrest medium for 48 h. Cells were labeled with [^3^H]thymidine for the final 8 h of the 48-h incubation period. Total cell lysates were harvested and mixed with scintillation cocktail, and [^3^H]thymidine incorporation (cpm) was measured using a liquid scintillation counter. 5-Bromo-2′-deoxyuridine (BrdU) incorporation was assayed according to the manufacturer's protocols (EMD Millipore). Briefly, RASMCs were seeded at 2 × 10^3^ cells/well in a 96-well plate, incubated for 48 h, and, during the last 8 h of the incubation period, labeled with BrdU. Cells were then fixed and incubated with anti-BrdU antibody (1:100 dilution) for 1 h in multiwell plates, washed, and incubated with a horseradish peroxidase-conjugated secondary antibody for 30 min. Colorimetric absorbance was measured in each well with a spectrophotometer (SpectraMax 190, Molecular Devices, Sunnyvale, CA) at dual (450 and 540 nm) wavelengths.

#### Fluorescence microcytometry.

For cell cycle stage analysis, VSMCs were seeded at a density of 7.5 × 10^4^ cells/well in six-well plates. After adenovirus transduction, cells were harvested at the indicated times. Cells were then washed with PBS and fixed in ice-cold 70% ethanol. Fixed cells were assessed for cell cycle stage using Cell Cycle Reagent (EMD Millipore) for 30 min, and DNA content was measured using a fluorescence microcytometer (Guava AFP96, EMD Millipore). DNA content of the measured cell populations (15,000 events, *n* ≥ 5 per condition) was categorized according to cell cycle stages using a cell cycle analysis program (EMD Millipore).

#### Morphological assessment of apoptosis.

Quiescent VSMCs were transduced with Ad/LacZ or Ad/Irx5 at the indicated MOI and switched to growth medium. At 30 h after transduction, the cells were incubated with 10 μg/ml Hoechst 33342 for 20 min for staining of nuclear DNA. Phase-contrast and epifluorescence images were captured using an inverted microscope (Olympus IX71, C Squared, Nashville, TN) at ×40 magnification.

#### Caspase activity assay.

Caspase-3 activity was measured using the CaspACE Assay System, Colorimetric (Promega, Madison, WI). Briefly, VSMCs were plated at 1 × 10^6^ cells per 90-mm dish. After serum deprivation, cells were treated for 6 h with Ad/LacZ, Ad/Irx5, or mock adenovirus. After removal of the virus, cells were cultured in growth medium. At the indicated times, detached and adherent cells were harvested. The cell lysates were centrifuged at 15,000 *g* for 20 min at 4°C. The cleared supernatant was transferred to a new microcentrifuge tube for measurement of total protein concentration and diluted to the same concentration. The assay was performed in a total volume of 100 μl in a 96-well plate. For measurement of caspase-3 activity, 20 μl of the lysate (100 μg of total protein) from each sample were used. The plate was incubated at 37°C for 4 h. Absorbance at 405 nm in the wells was measured using the spectrophotometer (SpectraMax 190, Molecular Devices). The rate of color change was normalized to the protein concentration. In some experiments the cell lysates were incubated with the caspase-3 inhibitor Z-DEVD-FMK (50 μmol/l; EMD Millipore) before assessment of caspase activity.

#### Statistical analysis.

Data reflect independent experiments that were performed at least three times with similar results. Values are means ± SD. Data were evaluated by *t*-test or one-way ANOVA. When the overall *F* test of the ANOVA was significant, a multiple-comparison (Tukey's) test was applied to determine the sources of differences, which were considered statistically significant when *P* ≤ 0.05.

## RESULTS

### 

#### Irx5 is expressed in cells of the peripheral vasculature under mitogenic conditions.

**Fig. 1.**
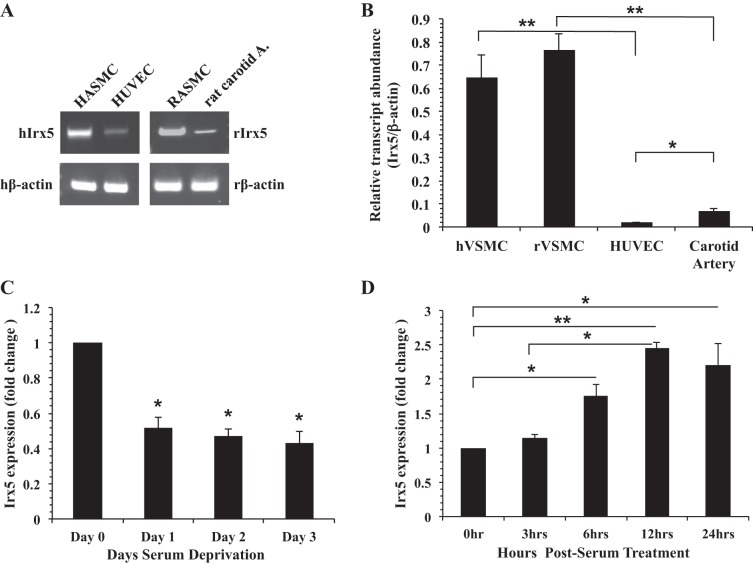
Iroquois homeobox transcription factor (*Irx5*) expression in peripheral vasculature and under mitogenic conditions. *A*: representative images of RT-PCR products from human aortic smooth muscle cells (HASMCs), human umbilical vein endothelial cells (HUVECs), rat aortic smooth muscle cells (RASMCs), and carotid artery tissue were resolved in 2% agarose gel, and expected-size bands were observed and compared with β-actin loading control [human and rat β-actin (hβ-actin and rβ-actin)]. hIrx and rIRx5, human and rat *Irx5*. *B*: quantitative RT-PCR assessment of endogenous *Irx5* expression in human and rat vascular smooth muscle cells (hVSMCs and rVSMCs), HUVECs, and carotid artery tissue. Data are expressed as fold change vs. β-actin. *C* and *D*: primary RASMCs were serum-deprived for 0–3 days or cultured under normal growth conditions for 0–24 h. Endogenous *Irx5* expression was determined by quantitative RT-PCR, normalized to β-actin, and expressed as fold change vs. *day 0* or 0 h. *P < 0.05; **P < 0.01.

To determine the presence of *Irx5* mRNA in the vasculature, we used RT-PCR to examine endogenous *Irx5* expression in cultured VSMCs, endothelial cells, and adult rat artery. Results in Fig. 1*A* demonstrate that *Irx5* mRNA was present in cultured HASMCs, HUVECs, RASMCs, and intact rat carotid arteries. To determine the relative expression level of *Irx5* in the vasculature, we examined *Irx5* expression in cultured VSMCs, endothelial cells, and adult rat carotid artery by quantitative RT-PCR. Our results (Fig. 1*B*) demonstrate that *Irx5* mRNA was present in cultured human VSMCs, HUVECs, rat VSMCs, and rat carotid arteries. Moreover, the expression level of *Irx5* was significantly higher in VSMCs than vascular endothelial cells and carotid arteries. It is well known that the proliferative capacity of adult VSMCs under normal conditions is reduced in vivo; we examined the expression of *Irx5* in the context of serum deprivation or stimulation in RASMCs. Results in Fig. 1*C* indicate that expression of endogenous *Irx5* in rat VSMCs was significantly decreased by serum deprivation after 24 h and was maintained over a period of 3 days. Addition of serum resulted in a significant increase in *Irx5* expression (Fig. 1*D*) as early as 6 h after serum stimulation, which was maintained over a period of 24 h.

#### IRX5 protein signal is increased in the neointima during proliferative vascular remodeling.

**Fig. 2.**
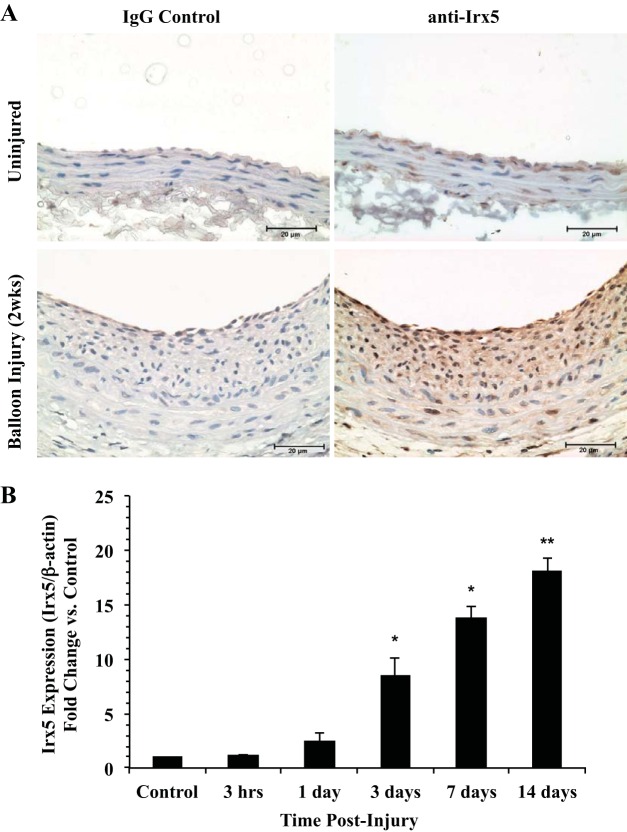
IRX5 immunohistochemistry during proliferative vascular remodeling. *A*: representative images of IRX5 immunostaining in balloon-injured and uninjured control rat carotid arteries show increased IRX5 staining in VSMCs in the neointima 14 days postinjury. IRX5-positive staining is present in medial, neointimal, and endothelial layers in injured arteries at 14 days postinjury; some background staining is present in the cytoplasm. No significant staining was observed in IgG control vessels. IRX5 and IgG primary antibody dilution was 1:600. Cell nuclei were counterstained with hematoxylin. Magnification ×40; scale bars = 20 μm. *B*: quantitative RT-PCR assessment of *Irx5* expression in total RNA isolated from balloon-injured rat carotid arteries (*n* ≥ 5) at 0–14 days postinjury. Data indicate a significant increase in *Irx5* expression as early as 3 days postinjury. *Irx5* expression was normalized to β-actin and is shown as fold change vs. uninjured control. *P < 0.05; **P < 0.01 vs. control.

To examine IRX5 protein in the context of vascular injury, adult rat carotid arteries were subjected to balloon catherization injury and examined for the presence of IRX5 protein by immunohistochemistry after 14 days. Results in Fig. 2*A* indicate positive staining for IRX5 in a population of VSMCs in the medial layer of uninjured arteries. However, we observed markedly elevated positive staining for IRX5 in VSMCs within the neointima, medial layer, and endothelium in injured arteries compared with uninjured control arteries. Notably, IRX5-positive staining was observed mainly within the nuclei of cells located in the neointima. However, some background staining was also observed in the cytoplasmic compartment in the medial layer of injured and uninjured arteries. No significant staining was observed in arteries stained with IgG2a isotype control antibody. To examine the temporal expression pattern of *Irx5* during proliferative vascular remodeling, we performed quantitative RT-PCR experiments using RNA samples extracted from balloon-injured rat carotid arteries at 3 h to 7 days postinjury. Our results demonstrate a significant increase in *Irx5* expression as early as 3 days (Fig. 2*B*), which was maintained at 3–14 days postinjury compared with uninjured contralateral carotid arteries (control). No significant difference in *Irx5* temporal expression was observed in control arteries over the 14-day period. Taken together, these results demonstrate that *Irx5* mRNA and IRX5 protein are present in the peripheral vasculature in vitro and in vivo and are significantly increased during VSMC proliferative remodeling.

#### Irx5 promotes cellular DNA synthesis in VSMCs.

**Fig. 3.**
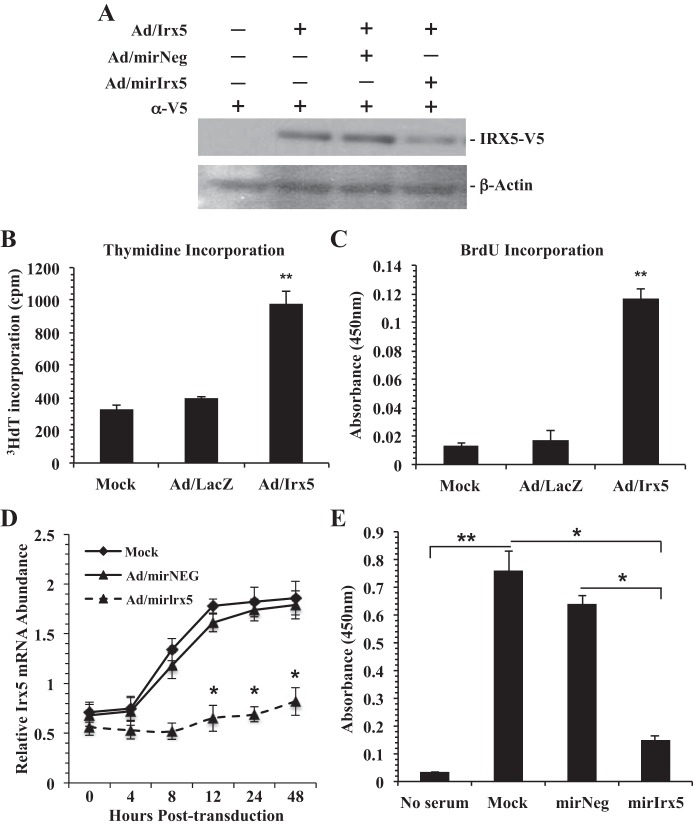
IRX5 promotes DNA synthesis. *A*: Western immunoblot of V5 epitope-tagged IRX5. RASMC protein lysates were harvested 48 h posttransduction, and Western blot analysis was performed with anti-V5 antibody (1:5,000 dilution). Transduction with Ad/Irx5 [multiplicity of infection (MOI) = 200] alone or with Ad/miR-Irx5 (MOI = 100) results in overexpression or knockdown, respectively, of IRX5-V5 fusion protein. Anti-rat β-actin (1:5,000 dilution) was used as loading control. *B* and *C*: Ad/Irx5 expression in quiescent RASMCs results in a significant increase in DNA synthesis compared with control conditions. RASMC were incubated in serum-deprived medium for 48 h and labeled with [^3^H]thymidine or 5-bromo-2′-deoxyuridine (BrdU) during the last 8 h. *D* and *E*: in RASMCs, transduction with Ad/miR-Irx5 (MOI = 200) under normal growth conditions results in a significant reduction of endogenous *Irx5* expression (*D*) and BrdU incorporation (*E*). No significant differences were observed in control conditions. *P < 0.05; **P < 0.01.

To investigate IRX5 function in VSMCs, we generated gain-of-function (Ad/Irx5) and loss-of-function (Ad/miR-Irx5) recombinant adenovirus vectors. RASMCs were transduced (MOI = 200) with Ad/Irx5-expressing mouse IRX5 with a conditional V5 fusion epitope located at the COOH-terminal region. Results in Fig. 3*A* demonstrate adenovirus vector-mediated ectopic IRX5 expression after 48 h, which was partially inhibited upon cotransduction with Ad/miR-Irx5 (MOI = 100). Previous reports indicate a role for *Irx5* in cell cycle regulation and apoptosis. Therefore, we investigated IRX5 function in DNA synthesis. RASMCs were transduced with Ad/Irx5, Ad/LacZ, or mock adenovirus. Cells were then serum-deprived for 48 h, with addition of [^3^H]thymidine during the last 8 h. Results indicate that [^3^H]thymidine incorporation and BrdU incorporation (Fig. 3, *B* and *C*) were significantly increased in Ad/Irx5-transduced VSMCs compared with VSMCs transduced with Ad/LacZ control and mock adenovirus.

Next, we performed transient *Irx5* knockdown studies using a synthetic microRNA targeted to endogenous *Irx5* mRNA (Ad/miR-Irx5) to examine the effects on DNA synthesis in RASMCs. Our results (Fig. 3*D*) indicate a significant reduction of *Irx5* mRNA abundance in RASMCs transduced with Ad/miR-Irx5 as early as 12 h posttransduction compared with Ad/miR-Neg vector and mock control conditions. Similarly, we observed a significant reduction of BrdU incorporation (Fig. 3*E*) in Ad/miR-Irx5-transduced cells compared with Ad/miR-Neg- and mock-transduced cells in normal growth conditions. These data indicate a functional role of IRX5 during DNA synthesis in VSMCs during quiescence and during proliferation in vitro.

#### Ectopic Irx5 expression promotes S-phase entry.

**Fig. 4.**
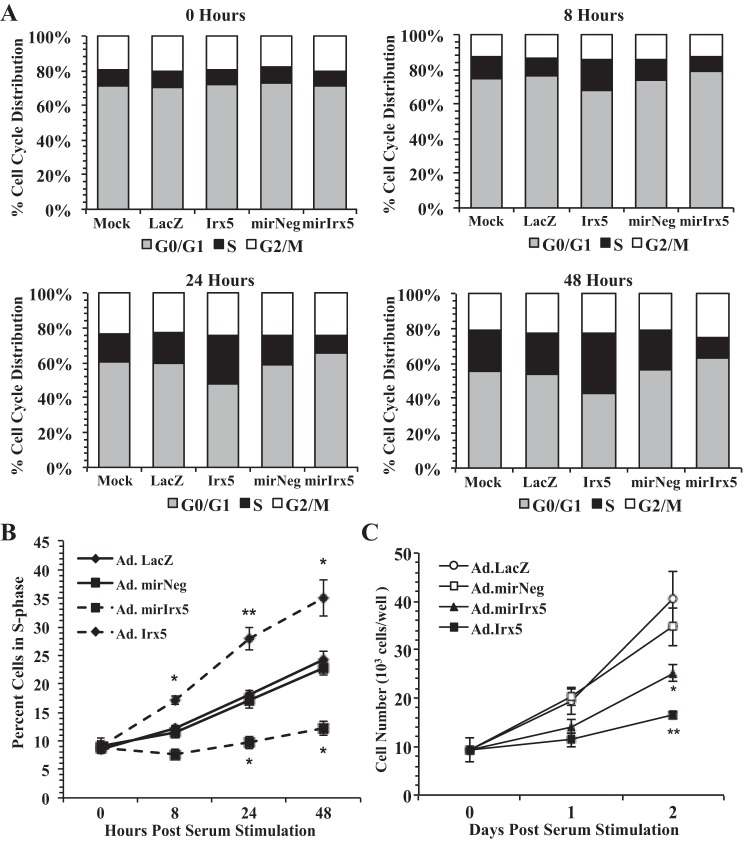
IRX5 increases the population of VSMCs in the S phase under normal growth conditions. Genetic gain or loss of *Irx5* modulates the percentile population of RASMCs in the S phase of the cell cycle. RASMCs were transduced with adenovirus vector (MOI = 200) and cultured under normal growth conditions. Cells were harvested, and cell cycle DNA content was analyzed for percent cell population at all stages of the cell cycle (*A*), percent S-phase population at 0–48 h (*B*), and number of viable RASMCs in each condition (*C*). *P < 0.05; **P < 0.01 vs. control vector.

Our previous results indicate that ectopic expression of *Irx5* results in an increase in DNA synthesis; therefore, we performed cell cycle analysis to determine if IRX5 function affects cell cycle stage distribution under mitogenic conditions. RASMCs were transduced (MOI = 200) with Ad/Irx5 or Ad/miR-Irx5 or the control vector Ad/LacZ or Ad/miR-Neg, respectively, for 8 h. Cells were growth-arrested for 24 h and then cultured in normal growth conditions for 48 h. Results from cell cycle analysis (Fig. 4*A*) indicate that cells transduced with Ad/Irx5 transitioned from the G_1_ to the S phase as early as 8 h. Moreover, RASMCs in the S-phase were maintained at significantly higher levels (Fig. 4*B*) at 8–48 h compared with mock- and Ad/LacZ-transduced cells. Transient knockdown of endogenous *Irx5* in RASMCs transduced with Ad/miR-Irx5 resulted in a significant reduction of the S-phase population at 24–48 h compared with Ad/miR-Neg-transduced cells. Interestingly, the proportion of RASMCs in the G_2_/M phase was not significantly different in any of the adenovirus vector-transduced conditions compared with mock-transduced cells. Viable cell count analysis (Fig. 4*C*) indicates a significant reduction of *Irx5-*transduced cells compared with control LacZ-transduced RASMCs. Transient knockdown of endogenous *Irx5* resulted in a significant reduction of cell numbers compared with control miR-Neg-transduced cells.

#### IRX5-induced S-phase entry is mediated by repression of P27^Kip1^ and CDK2 activation.

**Fig. 5.**
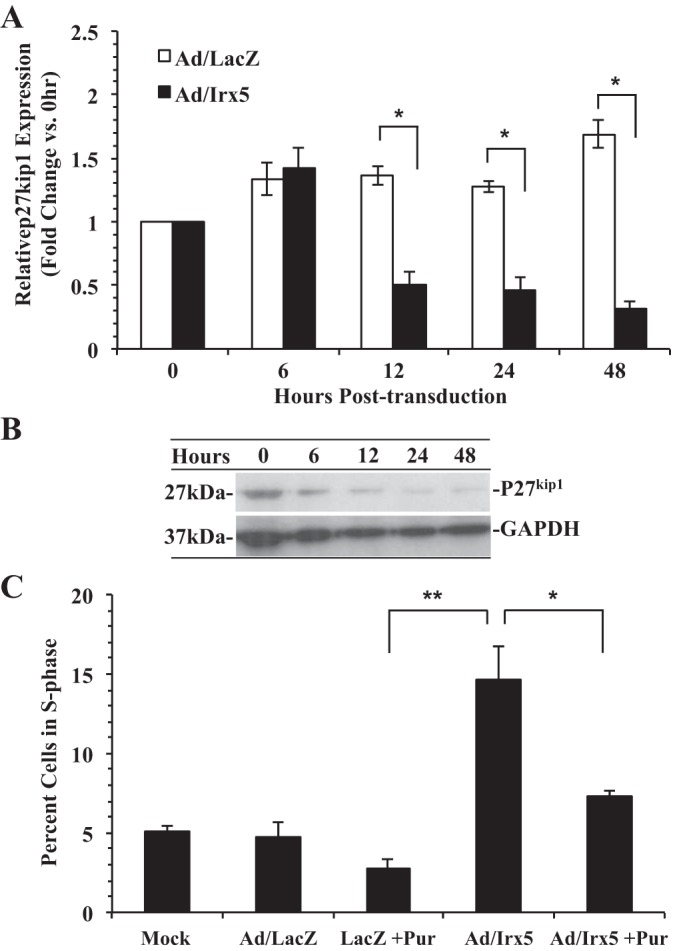
Effect of IRX5 on the G_1_/S-phase checkpoint control mediator P27^Kip1^. *A*: quantitative RT-PCR assessment of *p27*^*kip1*^ expression in quiescent RASMCs transduced with Ad/LacZ or Ad/Irx5 (MOI = 200). Total RNA was isolated from transduced VSMCs at 0–48 h after 48 h of quiescence. Relative *p27*^*kip1*^ mRNA abundance was normalized to β-actin and is shown as fold change vs. 0 h. *B*: Western immunoblot detection of P27^Kip1^ protein expression at 0–48 h posttransduction. *C*: the CDK2 inhibitor purvalanol A abrogates Ad/Irx5-mediated S-phase entry. Quiescent RASMCs were transduced with the indicated adenovirus (MOI = 200) in the presence of purvalanol A (Pur, 35 nM) or vehicle for 48 h. *P < 0.05; **P < 0.01 vs. control.

The CDK2 inhibitor P27^kip1^ has been well reported as a negative regulator of cell cycle progression at the G_1_/S-phase checkpoint (23). To examine the expression level of *p27*^*kip1*^ in *Irx5*-transduced cells, quiescent RASMCs were transduced with Ad/Irx5 or Ad/LacZ. At 0–48 h, total RNA was harvested and *p27*^*kip1*^ expression was examined by quantitative RT-PCR. Results in Fig. 5, *A* and *B*, demonstrate a significant reduction of *p27*^*kip1*^ expression and P27^Kip1^ protein levels in *Irx5*-transduced cells after 12 h compared with Ad/LacZ control conditions. Furthermore, pharmacological inhibition (Fig. 5*C*) of CDK2 activity with purvalanol A in Ad/Irx5-transduced RASMCs resulted in a significant reduction of cells in the S phase after 48 h compared with vehicle-treated cells. Therefore, these data suggest that ectopic expression of *Irx5* results in early repression of *p27*^*kip1*^ and subsequent loss of G_1_/S-phase cell cycle checkpoint control that is dependent on CDK2 activity.

#### Ectopic Irx5 expression results in an increase in the S-phase mediators E2f1 and Pcna1.

**Fig. 6.**
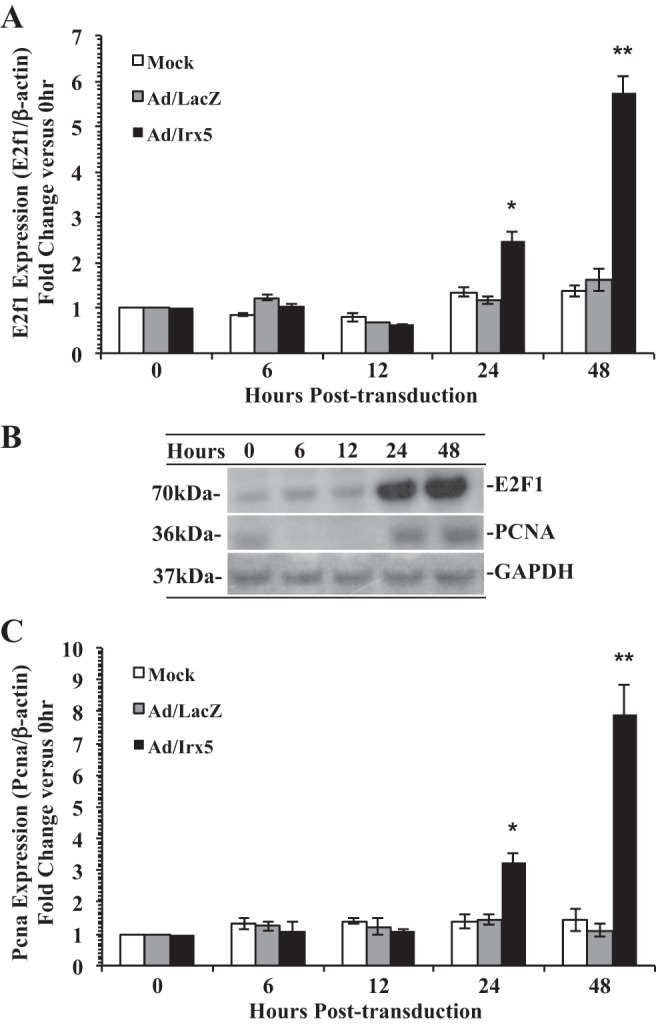
Effect of IRX5 on E2F transcription factor 1 (*E2f1*) and proliferating cell nuclear antigen (*Pcna*) cell cycle regulatory gene expression. Expression of *E2f1* and its target gene *Pcna* was elevated by *Irx5* in RASMCs. Quiescent cells were transduced by Ad/LacZ, Ad/Irx5 (MOI = 200), or mock adenovirus (PBS) and incubated in serum-deprived medium for 0–48 h. Cells were harvested at 0–48 h. *A* and *C*: assessment of *E2f1* and *Pcna* mRNA expression by quantitative RT-PCR, with β-actin used for normalization. *B*: Western blot analysis of E2F1 and PCNA protein levels. *P < 0.05; **P < 0.01 vs. control.

Previous reports have characterized E2F1 as an essential mediator of cell cycle progression in VSMCs (38, 39). Therefore, we examined the expression level of *E2f1* in RASMCs transduced with mock adenovirus, Ad/Irx5, or Ad/LacZ and incubated in serum-deprived medium for 48 h. Results demonstrate a significant increase in *E2f1* expression and E2F1 protein levels (Fig. 6, *A* and *B*) in cells transduced with Ad/Irx5 at 24 h posttransduction. Similarly, we observed a significant increase in *Pcna1* expression and PCNA protein (Fig. 6, *B* and *C*), a known gene target of *E2f1*, compared with Ad/LacZ and mock control conditions.

#### IRX5 induces an apoptotic phenotype in VSMCs.

**Fig. 7.**
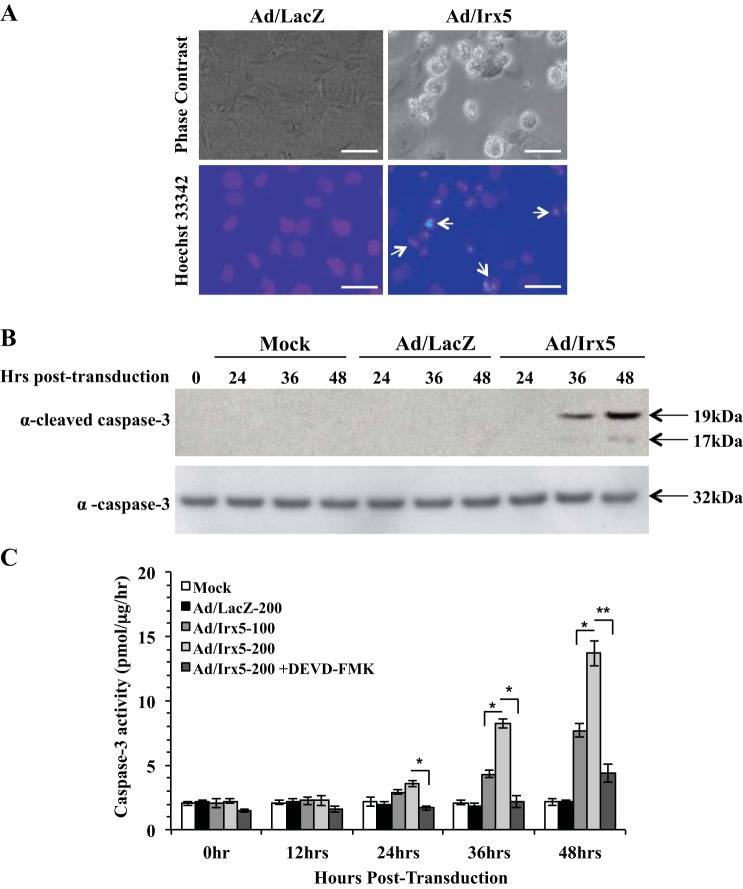
IRX5 induced the apoptotic phenotype in VSMCs. RASMCs were transduced with Ad/Irx5, Ad/LacZ (MOI = 200), or mock adenovirus (PBS) in serum-deprived medium for 6 h and then switched to normal growth medium for 0–48 h. *A*: transduced VSMCs were incubated in growth medium for 48 h. *Top*: phase-contrast image of vector-transduced RASMCs. *Bottom*: RASMCs treated with Hoechst 33342; bright-blue fluorescent masses of chromatin that cleaved and abutted at the nuclear membrane (arrows) were identified as apoptotic. *B*: cleaved caspase-3 in Ad/Irx5-transduced RASMCs. Transduced cells were harvested at 0–48 h, and cell lysates were analyzed by Western blotting using anti-cleaved caspase-3 antibody (17/19-kDa) and anti-full-length caspase-3 (35-kDa) antibody. *C*: increased caspase-3 activity in Ad/Irx5-transduced RASMCs. Cells were transduced at the MOI indicated. At 0–48 h, caspase-3 activity, normalized to protein content, was measured. Addition of the caspase-3 inhibitor Z-DEVD-FMK (40 μmol/l) resulted in suppression of the Ad/Irx5-induced increase in caspase-3 activity. Results are representative of ≥3 independent experiments. Magnification ×40; scale bar = 20 μm.

**Fig. 8.**
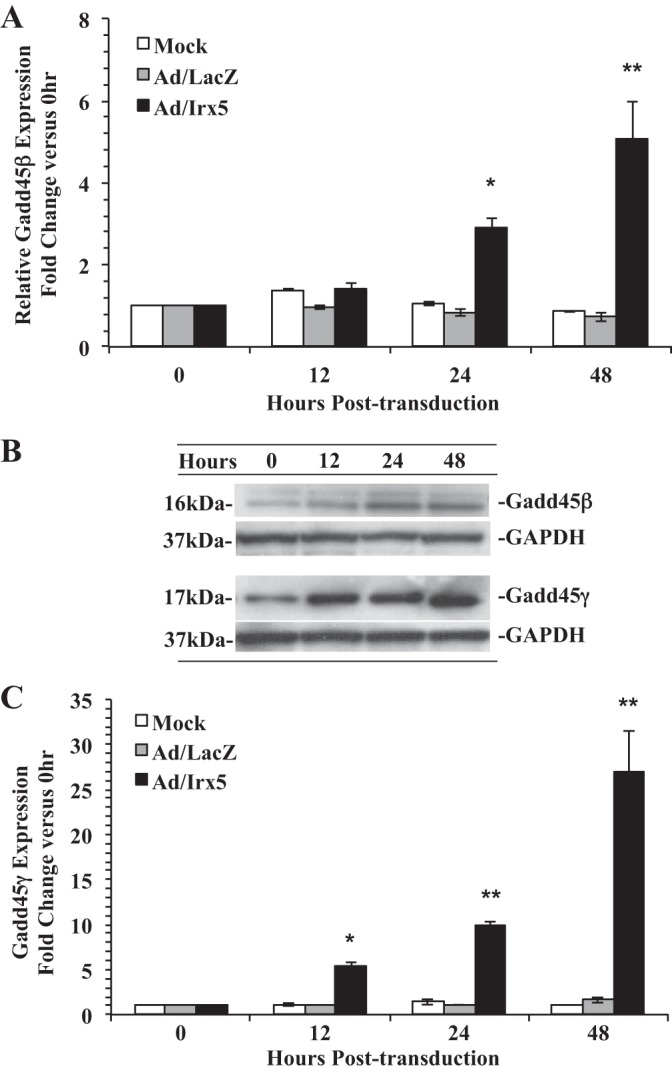
Effect of IRX5 on expression of DNA damage checkpoint control genes. *Gadd45β* and *Gadd45γ* mRNA (*A* and *C*) and protein (*B*) were dramatically increased in *Irx5*-transduced cells. Total RNA and protein were isolated from Ad/Irx5-, Ad/LacZ- (MOI = 200), or mock-transduced RASMCs under normal growth conditions at 0–48 h, and quantitative RT-PCR and Western immunoblotting were performed. *Irx5* relative expression levels were normalized to β-actin. Data are expressed as fold change vs. 0 h. *P < 0.05; **P < 0.01 vs. control vector.

Based on the observation that *Irx5*-transduced RASMCs resulted in an aberrant entry into the S phase, we examined if ectopic expression of *Irx5* also induces an apoptotic phenotype. To determine if cells were initiating apoptosis, we transduced quiescent RASMCs with Ad/Irx5 or Ad/LacZ and incubated them in normal growth medium. Morphological changes characteristic of cells undergoing apoptosis were observed, with cell rounding, shrinkage, cleavage, and condensation of chromatin by Hoechst 33342 staining after 48 h (Fig. 7*A*). Induction of apoptosis by *Irx5* was also evidenced by cleavage of procaspase-3, as observed by the appearance of the large (17/19-kDa) fragment of activated caspase-3 (Fig. 7*B*). To further evaluate apoptosis, we examined caspase-3 activity in RASMCs transduced with Ad/Irx5, Ad/LacZ, or mock adenovirus. Our results demonstrate that caspase-3 activity was significantly increased in a dose- and time-dependent manner (Fig. 7*C*) and that the caspase-3 inhibitor Z-DEVD-FMK (40 μmol/l) significantly inhibited IRX5-mediated caspase-3 activity. Moreover, RASMCs transduced with *Irx5* resulted in a significant increase in expression and protein levels of the DNA damage checkpoint control genes *Gadd45β* and *Gadd45γ* compared with control conditions (Fig. 8). Taken together, these results indicate a novel function of IRX5 to regulate cell cycle control at the G_1_/S-phase entry transition, which is demonstrated by a reduction of *p27^Kip1^* expression and CDK2 activation. Furthermore, we observed an IRX5-mediated increase in DNA synthesis and S-phase entry, with subsequent apoptosis, as indicated by elevated expression of the *Gadd45β* and *Gadd45γ* DNA damage repair genes.

## DISCUSSION

In recent years, molecular linkages between cell death, cell survival, and the cell cycle have become an object of intense research (23). The phosphoinositide 3-kinase/Akt pathway, cyclins, CDKs, CDK inhibitors, p53, retinoblastoma protein/E2F pathway, Myc family, and Bcl-2 family were reported to have partial cross-utilization in opposing cell physiological processes such as the cell cycle, apoptosis, and cell survival/proliferation. Here we report the novel function of *Irx5* to promote the G_1_/S-phase transition of the cell cycle, suppress proliferation, and, ultimately, induce apoptosis in adult VSMCs.

IRX5 has been previously reported to function in the embryonic development of different tissues and organs. Recent reports describe *Irx5* expression in the adult heart and functions to establish the cardiac ventricular repolarization gradient (9, 15, 34). We report, for the first time, *Irx5* expression in the mammalian vasculature in vitro and in vivo. Previous reports have characterized the homeodomain protein Gax as an antiproliferative mediator in VSMCs (44). In contrast, our data demonstrate that endogenous expression of *Irx5* is responsive to serum treatment, suggesting that *Irx5* may function as a proproliferative mediator. It will be interesting to examine in detail the function of IRX5 during proliferative vascular remodeling in vivo.

During our *Irx5* gain-of-function experiments, we observed a loss of G_1_/S-phase checkpoint control and aberrant induction of S-phase entry followed by apoptotic cell death in VSMCs. A recent study that characterizes transient depletion of *Irx5* in LNCaP cells reports blockade of the G_2_/M phase of the cell cycle followed by apoptosis (31). We also observed apoptosis in our experiments; however, we did not observe a significant increase in the population of cells in the G_2_/M phase. We also observed a significant reduction of the population of VSMCs in the S phase after transient knockdown of *Irx5* using a synthetic microRNA. In contrast, we did not observe a significant decrease in p21 or p53 expression in our *Irx5* gain-of-function studies. This may be due to differences in the growth characteristics of the cells utilized and/or the presence of cell-specific transcription cofactor expression in LNCaP epithelial cells compared with VSMCs. Indeed, *Irx5* null mice are viable, although they demonstrate a number of phenotypic differences compared with their wild-type counterparts.

Enforced expression of *Irx5* resulted in VSMC detachment and nuclear fragmentation, which, in combination with other characteristic markers, serve to distinguish apoptosis from cell senescence or inflammatory-necrotic modes of death (20, 37). To further substantiate the induction of apoptosis by *Irx5*, we examined the levels of cleaved caspase-3 and caspase-3 activity after transduction with *Irx5*. We found that overexpression of *Irx5* in VSMCs resulted in elevated levels of both cleaved caspase-3 formation and activity in a dose-dependent manner. Thus, *Irx5* appears to activate downstream effectors of apoptotic death. Of note, activation of caspases and cell death became apparent only after 36 h, which indicates that *Irx5* expression, entrance of the arrested VSMCs into the G_1_/S phase, and transition to the S phase are required before the cells can engage in the apoptosis signaling cascade. It is unclear if IRX5 directly or indirectly regulates expression of proapoptotic mediators and should be rigorously investigated.

Previous studies indicate that the vitamin D receptor can function as a cofactor in *Irx5*-mediated transcription regulation (31). Walker et al. (41) demonstrated that heterodimerzation of *Irx4* with the vitamin D receptor is essential for spatial repression of the slow myosin heavy chain 3 (*slow MyHC3*) gene in the ventricle of the adult heart. Costantini et al. (15) reported that the cardiac corepressor mBOP was required for *Irx5*-mediated repression of Kv4.2 K^+^ channel gene (*Kcnd2*) expression in ventricular cardiac myocytes. The presence of the cofactor mBOP in the endo- vs. epicardial layer of the heart is essential in the establishment of a proper epicardial K^+^ gradient function under normal conditions. Therefore, it is likely that other cell-specific cofactors may determine whether IRX5 functions to transactivate, or repress, target gene expression in vitro and in vivo. Furthermore, Wang et al. (42) reported that the hetero- and homodimerization of IRX proteins also function to modulate gene expression in vitro. These reports suggest that transcription cofactor(s) complexes and other histone modifier proteins such as histone deacetylases, may be required for *Irx5*-mediated induction of S-phase entry in VSMCs. However, cell-specific cofactors that may associate with IRX5 during VSMC proliferation remain to be determined.

Previous reports suggest that IRX proteins act mainly as transcriptional repressors that recognize and bind a unique palindromic motif (15, 18, 22). Thus the signaling mechanism of IRX5 effects may rely on other downstream target genes. Our results demonstrate that *Irx5*-induced S-phase entry was associated with a significant reduction of *p27*^*kip1*^ expression, which has been well reported to promote cell death through loss of G_1_/S-phase cell cycle checkpoint control (27, 38). Our experiments demonstrate that the CDK2 inhibitor purvalanol A blocked IRX5-induced S-phase entry, suggesting a possible IRX5 functional dependence on CDK2 activity through direct or indirect repression of *p27*^*kip1*^. Similarly, dysregulation of *E2f1* expression results in S-phase arrest and subsequent apoptosis mediated by p53 in many cell types, including VSMCs (7). Our additional results demonstrate that IRX5-induced S-phase entry and apoptosis were associated with upregulation of *E2f1* during mitogenic stimulation in VSMCs.

In the context of DNA damage checkpoint control, *Xenopus* Iroquois (XIRO) homeoproteins were reported to coordinate the cell cycle by regulating *Gadd45γ* expression in *Xenopus* (16). In a recent study using gene expression profiling, Rosati et al. (34) reported that increased levels of *Irx5* are associated with deregulation of the cell cycle and apoptosis mediators GADD45β, BRCA1, and Cdnk2a in the hearts of miRNA1-2 null mice, suggesting that regulation of these molecules was mediated by elevated *Irx5* expression. Similarly, we observed an *Irx5*-mediated induction of *Gadd45β* and *Gadd45γ* expression in aortic VSMCs. This observation indicates that the S-phase entry and apoptosis induced by *Irx5* in VSMCs are associated with induction of DNA damage. Whether *Irx5* can modulate expression of these genes directly, or indirectly, is unknown.

In the present study we demonstrate that *Irx5* is expressed in VSMCs. Forced expression of *Irx5* in adult VSMCs resulted in a loss of G_1_/S-phase checkpoint control, with subsequent apoptosis, in conjunction with modulated expression of cell cycle and DNA damage mediators. Given the critical role of VSMC growth and migration in proliferative vascular diseases, IRX5 may be an important target in the development of future therapeutic strategies to prevent postinjury vascular stenosis and other VSMC proliferative remodeling complications.

## GRANTS

This study was supported by National Institutes of Health Grants K01 HL-084760-01 (L. M. Anderson), 5R25 HL-003676 (to G. H. Gibbons), 1P50 HL-117929 (to H. A. Taylor), and G12 RR-003034 and the National Science Foundation through funding for Georgia Tech/Emory Center for the Engineering of Living Tissues (Grant EEC-9731643).

## DISCLOSURES

No conflicts of interest, financial or otherwise, are declared by the authors.

## AUTHOR CONTRIBUTIONS

D.L., V.P., and L.M.A. performed the experiments; D.L., V.P., and L.M.A. analyzed the data; D.L., M.B., and L.M.A. interpreted the results of the experiments; D.L. and L.M.A. prepared the figures; D.L., M.B., and L.M.A. drafted the manuscript; D.L., M.B., and L.M.A. edited and revised the manuscript; D.L., V.P., M.B., and L.M.A. approved the final version of the manuscript; M.B. and L.M.A. developed the concept and designed the research.

## References

[1] Abate-Shen C Deregulated homeobox gene expression in cancer: cause or consequence? Nat Rev Cancer 2: 777–785, 2002. 12360280 10.1038/nrc907

[2] Bao ZZ, Bruneau BG, Seidman JG, Seidman CE, Cepko CL Regulation of chamber-specific gene expression in the developing heart by Irx4. Science 283: 1161–1164, 1999. 10024241 10.1126/science.283.5405.1161

[3] Beisvag V, Lehre PK, Midelfart H, Aass H, Geiran O, Sandvik AK, Laegreid A, Komorowski J, Ellingsen O Aetiology-specific patterns in end-stage heart failure patients identified by functional annotation and classification of microarray data. Eur J Heart Fail 8: 381–389, 2006. 16753336 10.1016/j.ejheart.2006.05.004

[4] Bennett MR, Macdonald K, Chan SW, Boyle JJ, Weissberg PL Cooperative interactions between RB and p53 regulate cell proliferation, cell senescence, and apoptosis in human vascular smooth muscle cells from atherosclerotic plaques. Circ Res 82: 704–712, 1998. 9546379 10.1161/01.res.82.6.704

[5] Bilioni A, Craig G, Hill C, McNeill H Iroquois transcription factors recognize a unique motif to mediate transcriptional repression in vivo. Proc Natl Acad Sci USA 102: 14671–14676, 2005. 16203991 10.1073/pnas.0502480102 PMC1239941

[6] Bosse A, Stoykova A, Nieselt-Struwe K, Chowdhury K, Copeland NG, Jenkins NA, Gruss P Identification of a novel mouse Iroquois homeobox gene, Irx5, and chromosomal localisation of all members of the mouse Iroquois gene family. Dev Dyn 218: 160–174, 2000. 10822268 10.1002/(SICI)1097-0177(200005)218:1<160::AID-DVDY14>3.0.CO;2-2

[7] Bryja V, Pacherník J, Soucek K, Horvath V, Dvorák P, Hampl A Increased apoptosis in differentiating p27-deficient mouse embryonic stem cells. Cell Mol Life Sci 61: 1384–1400, 2004. 15170516 10.1007/s00018-004-4081-4 PMC11138945

[8] Bürglin TR Analysis of TALE superclass homeobox genes (MEIS, PBC, KNOX, Iroquois, TGIF) reveals a novel domain conserved between plants and animals. Nucleic Acids Res 25: 4173–4180, 1997. 9336443 10.1093/nar/25.21.4173 PMC147054

[9] Cai X Regulation of smooth muscle cells in development and vascular disease: current therapeutic strategies. Expert Rev Cardiovasc Ther 4: 789–800, 2006. 17173496 10.1586/14779072.4.6.789

[10] Chang MW, Barr E, Seltzer J, Jiang YQ, Nabel GJ, Nabel EG, Parmacek MS, Leiden JM Cytostatic gene therapy for vascular proliferative disorders with a constitutively active form of the retinoblastoma gene product. Science 267: 518–522, 1995. 7824950 10.1126/science.7824950

[11] Cheng CW, Chow RL, Lebel M, Sakuma R, Cheung HO, Thanabalasingham V, Zhang X, Bruneau BG, Birch DG, Hui CC, McInnes RR, Cheng SH The Iroquois homeobox gene, Irx5, is required for retinal cone bipolar cell development. Dev Biol 287: 48–60, 2005. 16182275 10.1016/j.ydbio.2005.08.029

[12] Christoffels VM, Habets PE, Franco D, Campione M, de Jong F, Lamers WH, Bao ZZ, Palmer S, Biben C, Palmer S, Harvey RP, Moorman AF Chamber formation and morphogenesis in the developing mammalian heart. Dev Biol 223: 266–278, 2000. 10882515 10.1006/dbio.2000.9753

[13] Christoffels VM, Keijser AG, Houweling AC, Clout DE, Moorman AF Patterning the embryonic heart: identification of five mouse Iroquois homeobox genes in the developing heart. Dev Biol 224: 263–274, 2000. 10926765 10.1006/dbio.2000.9801

[14] Cohen DR, Cheng CW, Cheng SH, Hui CC Expression of two novel mouse Iroquois homeobox genes during neurogenesis. Mech Dev 91: 317–321, 2000. 10704856 10.1016/s0925-4773(99)00263-4

[15] Costantini DL, Arruda EP, Agarwal P, Kim KH, Zhu Y, Zhu W, Zhu W, Lebel M, Cheng CW, Park CY, Pierce SA, Guerchicoff A, Pollevick GD, Chan TY, Kabir MG, Cheng SH, Husain M, Antzelevitch C, Srivastava D, Gross GJ, Hui CC, Backx PH, Bruneau BG The homeodomain transcription factor Irx5 establishes the mouse cardiac ventricular repolarization gradient. Cell 123: 347–358, 2005. 16239150 10.1016/j.cell.2005.08.004 PMC1480411

[16] De la Calle-Mustienes E, Glavic A, Modolell J, Gómez-Skarmeta JL Xiro homeoproteins coordinate cell cycle exit and primary neuron formation by upregulating neuronal-fate repressors and downregulating the cell-cycle inhibitor XGadd45γ. Mech Dev 119: 69–80, 2002. 12385755 10.1016/s0925-4773(02)00296-4

[17] Del Bene F, Wittbrodt J Cell cycle control by homeobox genes in development and disease. Semin Cell Dev Biol 16: 449–460 2005. 15840452 10.1016/j.semcdb.2005.02.001

[18] Gómez-Skarmeta J, de La Calle-Mustienes E, Modolell J The Wnt-activated Xiro1 gene encodes a repressor that is essential for neural development and downregulates Bmp4. Development 128: 551–560, 2001. 11171338 10.1242/dev.128.4.551

[19] Gomez-Skarmeta JL, Diez del Corral R, de la Calle-Mustienes E, Ferré-Marcó D, Modolell J Araucan and Caupolican, two members of the novel Iroquois complex, encode homeoproteins that control proneural and vein-forming genes. Cell 85: 95–105, 1996. 8620542 10.1016/s0092-8674(00)81085-5

[20] Gorski DH, LePage DF, Patel CV, Copeland NG, Jenkins NA, Walsh K Molecular cloning of a diverged homeobox gene that is rapidly down-regulated during the G_0_/G_1_ transition in vascular smooth muscle cells. Mol Cell Biol 13: 3722–3733, 1993. 8098844 10.1128/mcb.13.6.3722-3733.1993 PMC359848

[21] Gorski DH, Walsh K The role of homeobox genes in vascular remodeling and angiogenesis. Circ Res 87: 865–872, 2000. 11073881 10.1161/01.res.87.10.865

[22] He W, Jia Y, Takimoto K Interaction between transcription factors Iroquois proteins 4 and 5 controls cardiac potassium channel Kv4.2 gene transcription. Cardiovasc Res 81: 64–71, 2009. 18815185 10.1093/cvr/cvn259 PMC2721642

[23] Hiromura K, Pippin JW, Fero ML, Roberts JM, Shankland SJ Modulation of apoptosis by the cyclin-dependent kinase inhibitor p27^Kip1^. J Clin Invest 103: 597–604, 1999. 10074476 10.1172/JCI5461 PMC408127

[25] Jones FS, Meech R, Edelman DB, Oakey RJ, Jones PL Prx1 controls vascular smooth muscle cell proliferation and tenascin-C expression and is upregulated with Prx2 in pulmonary vascular disease. Circ Res 89: 131–138, 2001. 11463719 10.1161/hh1401.093582

[26] Jones PL Homeobox genes in pulmonary vascular development and disease. Trends Cardiovasc Med 13: 336–345, 2003. 14596950 10.1016/j.tcm.2003.09.001

[27] Kudoh T, Dawid IB Role of the iroquois3 homeobox gene in organizer formation. Proc Natl Acad Sci USA 98: 7852–7857, 2001. 11438735 10.1073/pnas.141224098 PMC35431

[28] Li X, Nie S, Chang C, Qiu T, Cao X Smads oppose Hox transcriptional activities. Exp Cell Res 312: 854–864, 2006. 16405960 10.1016/j.yexcr.2005.12.002

[29] Liu D, Hou J, Hu X, Wang X, Xiao Y, Mou Y, De Leon H Neuronal chemorepellent Slit2 inhibits vascular smooth muscle cell migration by suppressing small GTPase Rac1 activation. Circ Res 98: 480–489 2006. 16439689 10.1161/01.RES.0000205764.85931.4b

[30] McNeill H, Yang CH, Brodsky M, Ungos J, Simon MA mirror encodes a novel PBX-class homeoprotein that functions in the definition of the dorsal-ventral border in the *Drosophila* eye. Genes Dev 11: 1073–1082, 1997. 9136934 10.1101/gad.11.8.1073

[31] Myrthue A, Rademacher BL, Pittsenbarger J, Kutyba-Brooks B, Gantner M, Qian DZ, Beer TM The Iroquois homeobox gene 5 is regulated by 1,25-dihydroxyvitamin D3 in human prostate cancer and regulates apoptosis and the cell cycle in LNCaP prostate cancer cells. Clin Cancer Res 14: 3562–3570, 2008. 18519790 10.1158/1078-0432.CCR-07-4649

[32] Perović S, Schröder HC, Sudek S, Grebenjuk VA, Batel R, Stifanić M, Muller IM, Muller WE Expression of one sponge Iroquois homeobox gene in primmorphs from *Suberites domuncula* during canal formation. Evol Dev 5: 240–250, 2003. 12752763 10.1046/j.1525-142x.2003.03023.x

[33] Peters T, Dildrop R, Ausmeier K, Rüther U Organization of mouse Iroquois homeobox genes in two clusters suggests a conserved regulation and function in vertebrate development. Genome Res 10: 1453–1462, 2000. 11042145 10.1101/gr.144100 PMC310936

[34] Rosati B, Grau F, McKinnon D Regional variation in mRNA transcript abundance within the ventricular wall. J Mol Cell Cardiol 40: 295–302, 2006. 16412459 10.1016/j.yjmcc.2005.11.002

[35] Sata M, Perlman H, Muruve DA, Silver M, Ikebe M, Libermann TA, Oettgen P, Walsk K Fas ligand gene transfer to the vessel wall inhibits neointima formation and overrides the adenovirus-mediated T cell response. Proc Natl Acad Sci USA 95: 1213–1217, 1998. 9448311 10.1073/pnas.95.3.1213 PMC18722

[36] Schwartz SM, deBlois D, O'Brien ER The intima: soil for atherosclerosis and restenosis. Circ Res 77: 445–465, 1995. 7641318 10.1161/01.res.77.3.445

[37] Searle J, Kerr JF, Bishop CJ Necrosis and apoptosis: distinct modes of cell death with fundamentally different significance. Pathol Annu 17: 229–259, 1982. 7182752

[38] Shelat HS, Liu TJ, Hickman-Bick DL, Barnhart MK, Vida T, Dillard PM, Willerson JT, Zoldhelyi P Growth suppression of human coronary vascular smooth muscle cells by gene transfer of the transcription factor E2F-1. Circulation 103: 407–414, 2001. 11157693 10.1161/01.cir.103.3.407

[39] Stanelle J, Stiewe T, Rödicker F, Köhler K, Theseling C, Pützer BM Mechanism of E2F1-induced apoptosis in primary vascular smooth muscle cells. Cardiovasc Res 59: 512–519, 2003. 12909334 10.1016/s0008-6363(03)00392-4

[40] Theokli C, Morsi El-Kadi AS, Morgan R TALE class homeodomain gene Irx5 is an immediate downstream target for Hoxb4 transcriptional regulation. Dev Dyn 227: 48–55, 2003. 12701098 10.1002/dvdy.10287

[41] Walker NI, Harmon BV, Gobé GC, Kerr JF Patterns of cell death. Methods Achiev Exp Pathol 13: 18–54, 1988. 3045494

[42] Wang GF, Nikovits W Jr, Bao ZZ, Stockdale FE Irx4 forms an inhibitory complex with the vitamin D and retinoic X receptors to regulate cardiac chamber-specific slow MyHC3 expression. J Biol Chem 276: 28835–28841, 2001. 11382777 10.1074/jbc.M103716200

[43] Wright CV Vertebrate homeobox genes. Curr Opin Cell Biol 3: 976–982, 1991. 1687652 10.1016/0955-0674(91)90116-g

[44] Zhao Y, Ransom JF, Li A, Vedantham V, von Drehle M, Muth AN, Tsuchihashi T, McManus MT, Schwartz RJ, Srivastava D Dysregulation of cardiogenesis, cardiac conduction, and cell cycle in mice lacking miRNA-1-2. Cell 129: 303–317, 2007. 17397913 10.1016/j.cell.2007.03.030

